# Neutron scattering studies on dynamics of lipid membranes

**DOI:** 10.1063/5.0144544

**Published:** 2023-05-22

**Authors:** Michihiro Nagao, Hideki Seto

**Affiliations:** 1 National Institute of Standards and Technology Center for Neutron Research, Gaithersburg, Maryland 20899-6102, USA; 2 Department of Materials Science and Engineering, University of Maryland, College Park, Maryland 20742-2115, USA; 3 Department of Physics and Astronomy, University of Delaware, Newark, Delaware 19716, USA; 4 Institute of Materials Structure Science, High Energy Accelerator Research Organization, Tsukuba, Ibaraki 305-0801, Japan

## Abstract

Neutron scattering methods are powerful tools for the study of the structure and dynamics of lipid bilayers in length scales from sub Å to tens to hundreds nm and the time scales from sub ps to *μ*s. These techniques also are nondestructive and, perhaps most importantly, require no additives to label samples. Because the neutron scattering intensities are very different for hydrogen- and deuterium-containing molecules, one can replace the hydrogen atoms in a molecule with deuterium to prepare on demand neutron scattering contrast without significantly altering the physical properties of the samples. Moreover, recent advances in neutron scattering techniques, membrane dynamics theories, analysis tools, and sample preparation technologies allow researchers to study various aspects of lipid bilayer dynamics. In this review, we focus on the dynamics of individual lipids and collective membrane dynamics as well as the dynamics of hydration water.

## I. INTRODUCTION

Lipid molecules spontaneously form bilayers in water that are the basic building block of cell membranes. The bilayers define boundaries between cells and compartmentalize the cell into specialized organelles to accommodate various biological functions. These bilayers are, therefore, stable enough to maintain their structure yet soft and fluid enough such that embedded molecules move freely within the membranes. This unique balance of properties allows lipid bilayers to act as dynamic platforms, and the motions span a broad range of length and time scales. At long length and time scales, membranes deform, fuse, and undulate. Such membrane motions are collective movements of hundreds of self-assembled lipid molecules. By contrast, at short length and time scales, individual lipid molecules diffuse, rotate, and protrude within the membrane. This rich variety in lipid motions has direct implications for biological processes, and the individual and collective membrane fluctuations are controlled by the membrane properties.

**FIG. 1.**
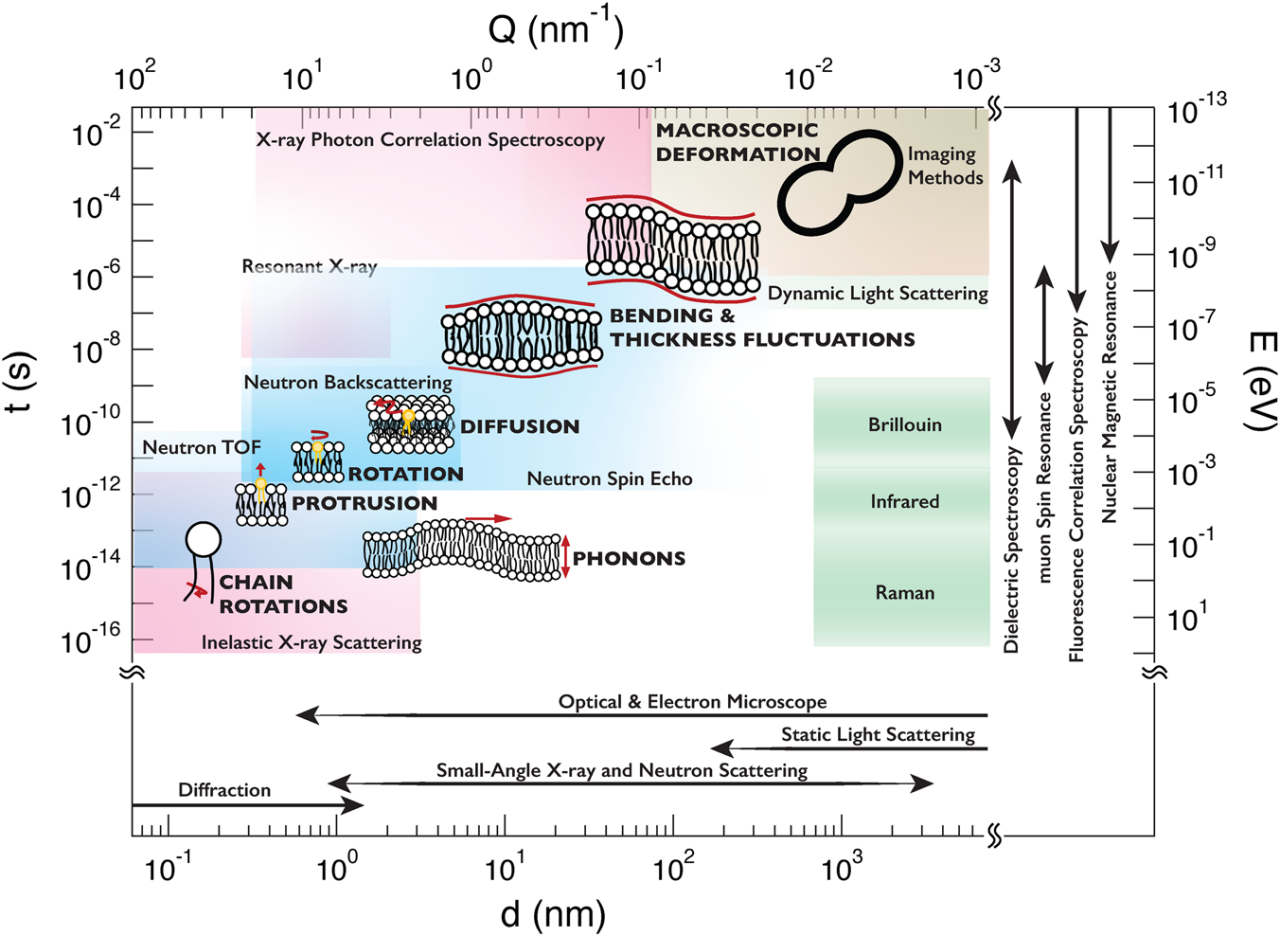
The length (*d*) and timescales (*t*), and the corresponding momentum (*Q*) and energy (*E*) transfers, covering the hierarchy of membrane dynamics. The range of interested length scales in lipid structure study is covered by a variety of scattering and microscopic techniques as shown in the bottom of the figure. The dynamic ranges of the spectroscopic techniques available to measure the different dynamics are also shown, with neutron techniques as light blue, x-ray in magenta, light in green, and fluorescence and optical imaging techniques in orange. Spectroscopic techniques, such as dielectric spectroscopy, muon spin resonance, nuclear magnetic resonance (NMR), and fluorescence correlation spectroscopy, access a broad range of timescales without any specific associated length scales. The lipid images in the figure is adapted with permission from Kelley *et al.*, *in Characterization of Biological Membranes: Structure and Dynamics*, edited by M.-P. Nieh, F. A. Heberle, and J. Katsaras (De Gruyter, 2019), pp. 131–176.^1^

Figure 1 summarizes lipid motions spanning a broad dynamic range together with different experimental techniques.^1^ Optical techniques cover length scales longer than the wavelength of light (several hundred nm) and the time scales longer than ms. However, it is important to note that recent developments in super-resolution optical techniques extend both the length and timescale resolutions of microscopy methods.^2^ Spectroscopic techniques, such as dielectric and nuclear magnetic resonance (NMR) spectroscopies, cover a broad range of time scales, but the timescale information is not readily associated with the length scale of the motions. Scattering techniques using light, x-ray, and neutron, on the other hand, access both length and time scales simultaneously, and the spatiotemporal range of the different techniques depends on the probe. Light scattering covers relatively long length scales while x-ray covers atomic to molecular length scales. Typically, the energy resolution (or observation time scales) is related to the incident energy of the probe, which is very high for x-rays (∼keV) and higher than the thermal energy of light (∼eV). On the other hand, thermal or cold neutrons have wavelengths close to the atomic to molecular scales and probe energies close to thermal energy (∼meV). Therefore, neutrons are good probes of the thermal fluctuations of atoms, molecules, and molecular assemblies. Given the power of neutron scattering tools for the study of lipid bilayers and the rapidly growing number of publications, there are several prior review papers that also discuss neutron scattering and lipid bilayers as well as introduce the properties of neutrons well.^3–8^

Among dynamic neutron scattering techniques, triple-axis and time-of-flight (TOF) spectrometers cover energy ranges typically in meV scales (sub ps in time scales), while backscattering (BS) accesses down to 1 *μ*eV scales (ns time scales) and neutron spin echo (NSE) techniques accesses tens of neV scales (*μ*s time scales). In lipid membrane dynamics studies, mostly BS and NSE techniques are used to access individual and collective lipid motions, such as rotation, translation, acyl tail motions, and collective membrane fluctuations. Some review papers focused on the dynamics of lipid molecules^5,7,9,10^ and collective membrane fluctuations^1,7,9–11^ have been published. Here, in this review, we mainly focus on the collective membrane fluctuations and how these experimental data are analyzed based on membrane dynamics theories. Section II introduces the theory of dynamic neutron scattering. Various examples that use NSE to study different types of bilayers composed of different types of lipids, various additives, or internal membrane organizations are given in Sec. III. Self-motions of individual lipid molecules as well as the hydration water dynamics are briefly reviewed in Sec. IV. Recent advances to measure collective acyl tail motions are given in Sec. V, and the paper closes with a summary and outlook for the field. Abbreviations used in this paper are listed at the end.

## II. THEORY OF DYNAMIC NEUTRON SCATTERING

### A. Basics of neutron scattering

The neutron is a subatomic particle that is neutral in charge and has a mass slightly greater than that of a proton, that is, a mass of each nucleon is approximately one atomic mass unit. A neutron is composed of three quarks, udd, and has a mean square radius of about $0.8\times 10^{-15}$ m, and it is a spin −1/2 fermion. This means that the neutron mainly interacts with the nucleus of an atom through the very short range nuclear force and is also influenced by the long range electromagnetic field within materials. Due to the particle-wave duality, one can define the de Broglie wavelength of neutrons *λ* as

$$\lambda =\frac{h}{m_{n}v},$$ (1)

where *h*, *m_n_*, and *v* are the Planck's constant, the neutron mass, and the speed of the neutron, respectively. In a neutron scattering experiment, the changes in momentum and energy are identified. By defining the wavenumber q=2π/λ and the initial and final momentum $m_{n}{\vec{v}}_{i}=\hbar {\vec{q}}_{i}$ and $m_{n}{\vec{v}}_{f}=\hbar {\vec{q}}_{f}$, respectively, the momentum conservation law is as follows:

$$\hbar \vec{Q}=\hbar ({\vec{q}}_{i}-{\vec{q}}_{f}),$$ (2)

where ℏ=h/2π is the reduced Planck's constant, and $\vec{Q}={\vec{q}}_{i}-{\vec{q}}_{f}$ is defined as the scattering vector, often also referred to as the momentum transfer vector. On the other hand, the energy conservation law is written as

$$E=\hbar \omega =E_{i}-E_{f}=\frac{\hbar^{2}}{2m_{n}}(q_{i}^{2}-q_{f}^{2}),$$ (3)

where *E* is the energy transfer, *ω* is the frequency of the exchanged energy, and *E_i_* and *E_f_* are the initial and final neutron energies, respectively. Here, we consider the magnitude of $\vec{Q}$ by defining the angle between ${\vec{q}}_{i}$ and ${\vec{q}}_{f}$ as the scattering angle, *θ_s_*, and applying $q_{i}\approx q_{f}$ ( ℏω≈0, i.e., assuming a small energy transfer) as

$$|\vec{Q}|=Q=2q_{i}\;\text{sin}\;\left(\frac{\theta_{s}}{2}\right)=\frac{4\pi }{\lambda }\text{sin}\;\left(\frac{\theta_{s}}{2}\right).$$ (4)

In the case of an ordered system, Bragg's law states that constructive interference appears when $2d\;\text{sin}\;\left(\frac{\theta }{2}\right)=n\lambda$, where *d* is the spacing between scattering planes and *n* is an integer. For such systems, *Q* is simply given as Q=2π/d, demonstrating the reciprocal relationship that large length scales are associated with small *Q* values and vice versa.

The primary aim of neutron scattering is to determine the probability of neutrons being scattered in $\vec{Q}$ with energy transfer ℏω, known as the dynamic structure factor (DSF), $S(\vec{Q},\omega )$, which is defined as

$$S(\vec{Q},\omega )=\frac{1}{N}\sum_{n,m}b_{n}b_{m}\int \left\langle e^{-i\vec{Q}\cdot \left\{{\vec{r}}_{m}(0)-{\vec{r}}_{n}(t)\right\}}e^{-i\omega t}dt\right\rangle ,$$ (5)

where ⟨⋯⟩ indicates an ensemble average over all pairs of atoms, and *N* is the number of atoms in the sample. The strength of the scattering is determined by the scattering length *b_n_* of the *n*th nucleus, and the spatial and time correlations between nuclei at positions ${\vec{r}}_{n}$ and ${\vec{r}}_{m}$ at time difference *t* are measured over a length scale determined by $\vec{Q}$.

The neutron scattering length that originates from the strength of the interaction between a neutron and the nucleus depends on the isotope of the element as well as the nuclear spin state. Due to the random distribution of the different types of nuclei in a material, the origin of the neutron scattering can be separated to two parts: the average neutron scattering length $\bar{b_{n}}\bar{b_{m}}$ and its difference from the mean ${\left\langle b_{n}\right\rangle }^{2}-{\bar{b_{n}}}^{2}$.^12^ The average neutron scattering gives distinct correlation between different nuclei, while the difference term gives self-correlations between the same nucleus. These terms are referred to as coherent and incoherent scattering, respectively, and the total DSF can be separated to coherent and incoherent DSFs as

$$S(\vec{Q},\omega )=S_{\text{coh}}(\vec{Q},\omega )+S_{\text{inc}}(\vec{Q},\omega ).$$ (6)

The coherent DSF, $S_{\text{coh}}(\vec{Q},\omega )$, represents mutual correlation of atoms and gives information on relative positions and motions of a couple of atoms, while the incoherent DSF, $S_{\text{inc}}(\vec{Q},\omega )$, represents self-correlation of atoms. Specifically, the incoherent scattering cross section, $\sigma_{\text{inc}}=4\pi ({\left\langle b_{n}\right\rangle }^{2}-{\bar{b}}_{n}^{2})$ for an atom *n*, of hydrogen (^1^H) is the largest of all the elements, and therefore, the incoherent scattering signal is often dominated by the self-motion of ^1^H in hydrogen containing materials. As the scattering lengths of hydrogen and deuterium are very different, one can selectively observe coherent (collective structure and dynamics) and incoherent (self-motion) components within the system by selecting molecules with different deuteration schemes (see Sec. II C).

Equation (5) contains the Fourier transform of the intermediate scattering function (ISF), $S(\vec{Q},t)$, with respect to *t* which is written as

$$S(\vec{Q},t)=\frac{1}{N}\sum_{n,m}b_{n}b_{m}\left\langle e^{-i\vec{Q}\cdot \left\{{\vec{r}}_{m}(0)-{\vec{r}}_{n}(t)\right\}}\right\rangle .$$ (7)

Therefore, both $S(\vec{Q},\omega )$ and $S(\vec{Q},t)$ contain the same information on the spatial and time correlations between a couple of atoms. Furthermore, the Fourier transform of the ISF in space represents the van Hove space–time correlation function, $G(\vec{r},t)$^13^

$$G(\vec{r},t)=\frac{1}{N}\sum_{n,m}b_{n}b_{m}\left\langle \delta \left(\vec{r}-{\vec{r}}_{m}(0)+{\vec{r}}_{n}(t)\right)\right\rangle .$$ (8)

This relation describes the probability of finding a particle at position ${\vec{r}}_{n}$ at time *t* given the same or another particle was at ${\vec{r}}_{m}$ at time 0, and the correlation function describes all of the equilibrium physics of the system. Scattering experiments, therefore, directly access either $S(\vec{Q},\omega )$ or $S(\vec{Q},t)$ that describes the equilibrium structure and dynamics of the system.

In a static scattering experiment such as small-angle neutron scattering (SANS), we do not specify the energy exchanged between the neutrons and the samples and integrate the scattering over all exchanged energies

$$\begin{array}{c} S(\vec{Q})=\int S(\vec{Q},\omega )d\omega \\ =\int (S_{\text{coh}}(\vec{Q},\omega )+S_{\text{inc}}(\vec{Q},\omega ))d\omega \\ =S_{\text{coh}}(\vec{Q})+S_{\text{inc}}(\vec{Q}), \end{array}$$ (9)

which corresponds to the Fourier transform of the instantaneous spatial atomic correlations in the system, i.e., the structure of the sample. It is noted that $S_{\text{inc}}(\vec{Q})$ is a constant, independent of $\vec{Q}$, and therefore considered as a background in static structure measurements. However, this is not the case for quasi-elastic neutron scattering (QENS), where $\int S_{\text{inc}}(\vec{Q},\omega )d\omega$ is calculated in a limited energy range and considered as elastic incoherent structure factor (EISF), which is not a constant with respect to $\vec{Q}$ and depends on the geometrical constraints in which the atoms of interest are situated.^14^ Hereafter, we only consider isotropic scattering cases for simplicity and treat the vector $\vec{Q}$ as a scalar *Q*.

### B. Inelastic/quasi-elastic neutron scattering techniques

Measuring the DSF, S(Q,ω), requires keeping track of the energy exchanged between the neutrons and the sample ( ℏω) that are scattered at a defined angle (*θ_s_* or corresponding *Q*). This type of measurement is called inelastic or quasi-elastic neutron scattering (INS or QENS). INS typically refers to scattering originating from oscillatory motions with a specific timescale and energy transfer and therefore reflects excitation processes in a sample. The S(Q,ω) shows a characteristic peak at a finite energy transfer *ω_p_*, which follows a dispersion relationship in the (Q,ω) plane. Meanwhile, QENS originates from motions that have a range of relaxation times and associated energy transfers and therefore reflects relaxation processes in a sample. In this case, the S(Q,ω) is characterized as a broadening of the elastic peak around ω≈0, where the degree of the broadening depends on *Q*. Most spectrometers determine the energy exchanged by analyzing the initial and final neutron energies. Neutron BS and TOF instruments are capable of resolving 1–100 *μ*eV changes in neutron energy, providing dynamics information on picosecond to nanosecond time scales.^15,16^ These techniques mostly measure the incoherent or “self” dynamics—the correlations between the relative positions of a given atom at different times—by selecting appropriate *Q* values and are very useful for determining the diffusion of hydration water, lipids, and even other small molecules embedded in lipid membranes.

As collective membrane fluctuations are much slower than the individual molecular motions, an improved energy resolution spectroscopy is necessary. The NSE technique, which is the highest energy resolution neutron spectroscopy technique,^17^ has been developed to measure relaxation times up to 1 *μ*s.^18^ It is noted that NSE is mostly applied to measuring coherent dynamics—dynamics that originate from correlations between relative positions of different atoms at different times—or the collective dynamics in lipid membranes. Though the number of examples is limited, NSE has been used to measure self-motions of lipid molecules.^19^ This technique also directly measures the ISF, *S*(*Q*, *t*), instead of S(Q,ω). Because NSE works in the time domain, it is best suited to measuring relaxation processes.

### C. Scattering length and scattering length density

Typically, when atomic scale information is extracted from neutron scattering experiments, the power of neutron scattering is expressed by the scattering length *b_n_* of the *n*th nucleus. However, when the observation length scales become longer, such as those observed at small scattering angles, individual atomic information is not as important, and instead, the density of scattering length is more important. As the energy resolution of the NSE technique is extended to 1 *μ*s, the relevant length scales are also extended to longer scales. Therefore, it is convenient to consider the scattering length density of a molecule instead of the scattering length of an atom. The scattering length density *ρ* of a molecule is defined as

$$\rho =\frac{1}{v_{m}}\sum_{j}^{k}b_{j},$$ (10)

where *b_j_* is the bound coherent scattering length of the *j*th atom in the molecule consisting of total *k* atoms with the molecular volume *v_m_*. Some calculation software are available for users, such as the one at NIST.^20^

For example, H_2_O and D_2_O scattering length densities vary by an order of magnitude and in sign, $-0.56\times 10^{-6}$ and $6.36\times 10^{-6}$ Å^−2^, respectively. When a protiated particle is dissolved in H_2_O, the scattering length density of the particle is close to that of H_2_O, and therefore, the coherent scattering intensity is not strong. However, if the same particle is dissolved into D_2_O, the difference in the scattering length density, i.e., the scattering contrast, is much larger, and we observe strong scattering signals originating from the particle. As such, we choose different deuteration schemes depending on what we want to observe in a neutron scattering experiment.

**FIG. 2.**
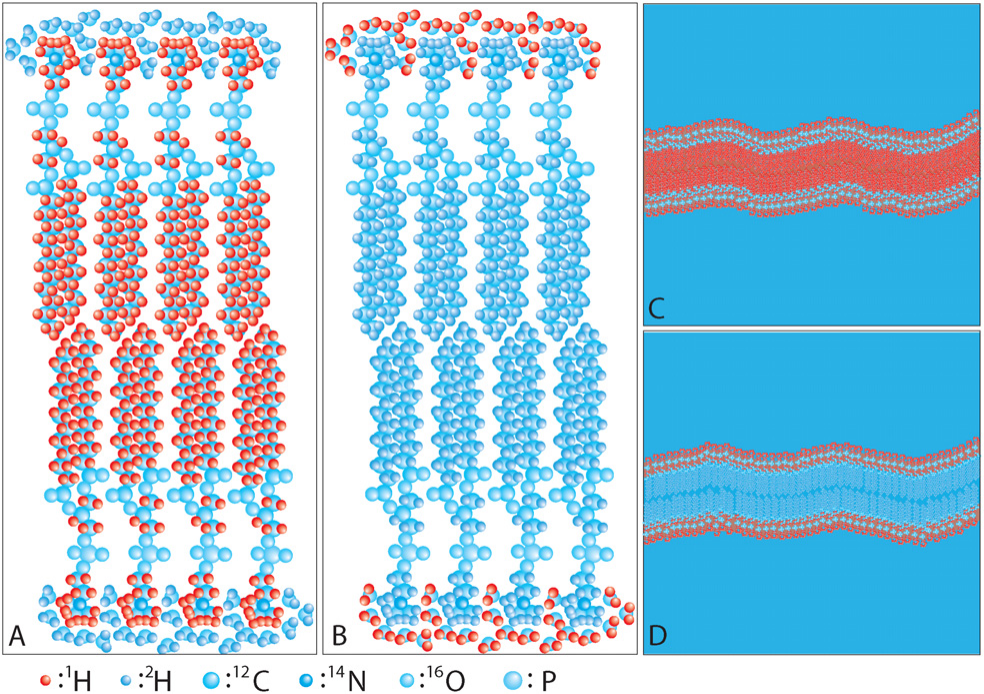
Schematic drawing of lipid and water molecules and neutron scattering lengths. Different atoms have different neutron scattering lengths, which are represented by different colors in the figure. (a) Lipid molecules are protiated while water is deuterated; a suitable deuteration scheme to measure lipid dynamics by QENS. (b) Lipid molecules are deuterated while water is protiated; a preferable deuteration scheme to measure water dynamics by QENS. (c) The same deuteration scheme as in A, while the membrane is seen in larger length scales for NSE; a deuteration scheme to see the membrane dynamics as a whole. (d) Lipid acyl tails are deuterated and dispersed in deuterium oxide solvent and observation length scales are relatively long; sensitive to internal membrane dynamics such as thickness fluctuations.

In the case of lipid bilayers, we may use the following schemes to observe different contributions from different components within the system. In a BS experiment, when protiated lipids are dissolved into D_2_O [see Fig. 2(a)], hydrogen motions in the lipids are the target of the observation, while water motions can be focused by employing a sample with perdeuterated lipids in H_2_O as shown in Fig. 2(b). In an NSE experiment, when protiated lipids are in D_2_O as shown in Fig. 2(c), the neutron scattering contrast provides the structure and dynamics of the whole bilayers. On the other hand, when tail-deuterated lipids are dissolved in D_2_O [Fig. 2(d)], internal membrane structure and dynamics are detailed as the scattering contrast is given to distinguish the head groups of the lipids.

## III. COLLECTIVE MEMBRANE DYNAMICS

### A. Membrane dynamics theories

The elastic properties of lipid bilayers were first examined with the successful curvature elasticity theory developed by Helfrich.^21^ He treated the membrane as a continuum and wrote the interfacial bending energy of the thin sheet as

$$F=\int \left[\frac{\kappa }{2}{\left(C_{1}+C_{2}-C_{s}\right)}^{2}+\bar{\kappa }C_{1}C_{2}\right]dS,$$ (11)

where *C*_1_ and *C*_2_ are the two principal curvatures of a membrane, *C_s_* is the spontaneous curvature, *κ* is the bilayer bending modulus, $\bar{\kappa }$ is the saddle-splay modulus, and the integration is over the area *S*. The saddle-splay modulus $\bar{\kappa }$ is sensitive to a topological change of the membrane. In certain cases, the topological change does not affect the membrane elastic energy, and the second term of Eq. (11) is negligible. As such, the two important parameters to determine the free energy of a membrane system are the spontaneous curvature *C_s_* and the bending modulus *κ*. The value of *κ* can be calculated either from static and dynamic properties.^22^ Here, we focus our attention on the dynamics of the membrane, where fluctuations are measured in nanosecond time scales.

Standard hydrodynamic theory predicts that the membrane's undulation fluctuation frequencies *γ* depend on the viscosity of the solvent *η*, the bending modulus *κ*, and the membrane undulation wavenumber $k(=2\pi /\lambda_{b})$ with *λ_b_* being the wavelength of the undulation fluctuations, as^23–25^

$$\gamma (k)=\frac{\kappa }{4\eta }k^{3}.$$ (12)

This dispersion relation implies that when the frequency of the undulation fluctuations is large (i.e., fluctuations are fast), the value of *k* is large (shorter fluctuation wavelengths). When *κ* is large, the frequency increases, meaning the fluctuations are faster, while large solvent viscosity *η* makes the undulation slower as the resistance to the membrane motions is larger.

The ISF, *S*(*Q*, *t*), of single membrane undulation fluctuations based on the Helfrich's interfacial bending energy was first calculated by Zilman and Granek (ZG) in 1996.^26,27^ They proposed that the ISF decays as a stretched exponential function with the stretching exponent of 2/3

$$S(Q,t)\propto \;\text{exp}\;\left[-{\left(\Gamma_{ZG}t\right)}^{2/3}\right]$$ (13)

and the relaxation rate Γ_*ZG*_ is proportional to *Q*^3^ as

$$\Gamma_{ZG}=0.025\gamma_{o}\sqrt{\frac{k_{B}T}{\tilde{\kappa }}}\frac{k_{B}T}{\eta }Q^{3},$$ (14)

where *γ_o_* is a coefficient that depends on the value of the effective bending modulus $\tilde{\kappa }$, and $\gamma_{o}=1$ when $\tilde{\kappa }$ is large enough, which is often the case for the lipid bilayers, and *k_B_* and *T* are the Boltzmann constant and absolute temperature, respectively. The validity of the theory was experimentally verified by dynamic light scattering (DLS)^28^ and NSE.^29,30^ Since then, this model is a standard tool used to analyze quasi-elastic scattering data from membranes composed of either surfactants or lipids.

For the case of NSE measurements, it is known that the estimated bending modulus from the ZG framework gives larger values than expected. In early studies, researchers used an effective solvent viscosity *η_eff_*, 3–4 times larger than solvent viscosity instead of *η* to extract reasonable values of *κ*. The hypothesis for this correction was because the length and time scales covered by NSE are sensitive to the internal dissipation mechanisms, an additional source of friction from the solvent at a short length and time scales may affect the dynamics, as was first proposed for surfactant monolayer dynamics studies.^30,31^ It is noted that the artificial factor 3 is known to be avoided when complete expression of ZG formalism is calculated by integrating *S*(*Q*, *t*).^32–34^

In 2010, Watson and Brown (WB) proposed that NSE data analysis frameworks need to take the membrane internal dissipation into account and developed a theory to incorporate this contribution to the ZG framework.^35,36^ Because of the finite thickness of the membrane, bending results in the outer leaflet being stretched and the inner leaflet being compressed. Thus, as a membrane is bent, a density gradient is created between the inner and outer leaflets, which cannot be readily relaxed within a short time. The original theoretical prediction by Seifert and Langer (SL) indicated that this density mode creates additional contributions in the bending modulus as^37,38^

$$\tilde{\kappa }=\kappa +2d_{n}^{2}K_{m},$$ (15)

where *d_n_* is the height of the neutral surface from the bilayer center and *K_m_* is the monolayer area compressibility modulus, respectively. The neutral surface is defined as the surface at which the bending energy is independent of the stretching energy. The importance of this contribution to NSE data was already suggested in the first NSE paper for lipid bilayer dynamics in 1993,^39^ but a deeper understanding of the contributions from the internal dissipation appeared in 2010s and later.

The first application of the modification to the ZG framework was performed by Lee and co-workers,^40^ where they estimated the contribution of the second term in the $\tilde{\kappa }$ was about 10κ, or the effective bending modulus measured by NSE was about an order of magnitude larger than *κ*. Some following studies also suggested that the second term in the expression for $\tilde{\kappa }$ was about an order of magnitude larger,^41,42^ which confirmed that NSE data are quite sensitive to the internal dissipation. This is a unique feature of the application of the NSE technique to measure membrane dynamics as this type of internal membrane dissipation is not readily accessible to other techniques.

Independent of the development of WB,^35^ Arriaga and colleagues experimentally observed a crossover of the relaxation rate Γ from $\Gamma \propto Q^{3}$ to $\Gamma \propto Q^{2}$ depending on *Q*. The *Q*^2^ dynamics was attributed to the intermonolayer slipping mode proposed in the SL model.^43^ The slipping modes, or hybrid curvature-compression modes as Monroy *et al.* called them,^43–45^ relate to the crucial coupling between bending and lateral compression of the membrane, first pointed out by Yeung and Evans,^46^ and the theoretical dispersion relation was derived in the SL model.^37,38^ The hybrid mode is also referred to as a friction mode since it involves the frictional coupling between the two leaflets of the bilayer sliding past with each other. The friction modes relax as $\Gamma_{\text{hyb}}∼Q^{2}$ which was exactly the observed dynamics at relatively higher *Q* in the NSE experiment on unilamellar vesicles (ULVs) of POPC bilayers.^43^ They estimated the intermonolayer friction coefficient *b*, which determines the relaxation time of the friction mode, to be on the order of 10^9^ Pa s/m for the POPC bilayers. While the friction coefficient is one of challenging parameters to experimentally observe (see recent microfluidic experiment results by Anthony and colleagues^47^) NSE has been suggested as another tool to access the mode relating to the interleaflet coupling.

Mell and colleagues studied the friction mode further by comparing the dynamics of DMPC, POPC, and SMPC bilayers.^44^ They measured the NSE signal at room temperature where DMPC and POPC were in the fluid phase. While the friction mode was observed in the case of POPC bilayers, this mode was not certain in the DMPC bilayers. They speculated that molecular disorder favors acyl tail interdigitation in unsaturated POPC compared to the fully saturated DMPC that promotes free-sliding between the bilayers in the case of DMPC bilayers. Consequently, the relaxation is expected to be slower in POPC membranes, which may be the reason why the friction modes appeared in the NSE range for POPC. Furthermore, in the case of SMPC that the bilayers were in the gel phase, and the high coupling expected in the gel phase prevented the detection of the friction mode in the solid-like bilayers. Mell and colleagues discuss more details of NSE data analysis based on the hybrid curvature-compression modes in their papers.^45^

As it appears in Sec. III C in more detail, Farago^31,48^ and Nagao^49^ independently observed surfactant membrane thickness fluctuations, and later the same mode was confirmed in lipid bilayers.^41^ These studies stimulated a theoretical development to consider dynamics of thick membranes. Bingham, Smye, and Olmsted (BSO) proposed an asymmetric membrane theory that considers temporal variations of the bilayer thickness and three contributions in the lipid bilayer dynamics were found; the standard undulation mode, the intermembrane mode which is similar to the sliding mode of SL theory, and the peristaltic mode that corresponds to the thickness fluctuations.^50^ They concluded that thickness fluctuations are driven by the compressibility of the membrane and are damped due to solvent and membrane viscosities. They estimated the predicted thickness fluctuations relaxation time using membrane parameters in the literature, and the predicted relaxation time of ≈100 ns was consistent with the experimental observations by Woodka *et al.*^41^

Moleiro and colleagues studied the effects of pore forming protein p10 of *Bacillus subtilis* bacteriophage ϕ29 into the DOPC/DOPG/PAzPC membrane, where PAzPC is an oxidized lipid with the short saturated tail.^51^ They included permeation contributions into the Helfrich free energy and solved the dynamic structure factor including the permeability mode to the ZG framework. When pores make the membranes permeable, the hydrodynamic impedance of the bending fluctuations is controlled by membrane permeability and the ISF follows:

$$\frac{S(Q,t)}{S(Q,0)}\approx \;\text{exp}\;\left[-{\left(\Gamma_{p}t\right)}^{1/2}\right]$$ (16)

with the relaxation rate Γ_*p*_

$$\Gamma_{p}(Q)\approx 0.045\frac{k_{B}T}{\kappa }k_{B}TpQ^{4},$$ (17)

where *p* represents the permeability kinetic coefficient.^51^ As pore formation makes the membrane more dynamic, this theory predicts the membranes are softer upon pore formation. The model fit their NSE data well, and the obtained parameters were reasonable. Without considering the permeability mode, Kelley and colleagues also observed softening of the membranes during the broad melting transition of charged DMPG bilayers which was attributed to pore formation.^52^

At short time scales accessible to NSE, Gupta and colleagues observed a crossover from short time dynamics to the single membrane fluctuations at about 3 ns in the time dependent mean squared displacement.^9,53,54^ By combining computer simulations with their NSE data, they proposed that the short time dynamics reflected the lateral motions of lipids that were restricted to about the size of the molecule.^53^ Granek, on the other hand, proposed the experimentally observed short time dynamics were possibly due to thickness deformations of wavelengths shorter than the membrane half-thickness.^55^ He pointed out that his interpretation assumes Gaussian statistics, while Gupta's observation included non-Gaussian behavior. Later, Gupta and colleagues used additional data to show their original idea of the lipid motion is a valid explanation for the short time dynamics.^56^

More recently, Hoffmann raised a caution for both the short timescale dynamics as well as thickness fluctuations as these data might have been affected by the data quality.^34^ Also, the estimates of the dynamics parameters could be varied depending on the method of the analysis used,^57^ and there are ongoing active developments in the field to better understanding lipid membrane dynamics. It is also noted here that lipid molecules are known to have tilt degrees of freedom, which creates another dissipation mechanism that is characterized by tilt modulus.^58–62^ Although the tilt modulus has not been integrated into the NSE analysis framework, Nagle has shown that the tilt modulus affects the fluctuation amplitude and the value of *κ* measured by x-ray diffuse scattering (XDS).^63,64^ Given the similar length scales of the XDS and NSE experiments, the tilt modulus may also affect the membrane dynamics measured at short times with NSE, and future works should explore these potential effects. In Secs. III B–III I, we review some experimental observations of lipid membrane dynamics and the extracted membrane properties using the analysis frameworks developed to date.

### B. Undulation fluctuations in different membrane geometries

In early NSE studies, the membrane dynamics were measured for hydrated bilayer stacks on a substrate. In the first NSE study of lipid bilayer dynamics, Pfeiffer and colleagues^39^ prepared highly ordered stacks of DPPC bilayers at a high hydration level. The advantage of using stacked supported bilayers is the ability to specify the scattering vector so that undulations parallel to the membrane surface are selectively measured. The measured ISFs were explained by a single exponential function with the relaxation rate $\Gamma \propto Q^{2.5}$. The exponent was in between a free membrane ( $\Gamma \propto Q^{3}$) and membrane stack as a two-component smectic-A liquid crystal ( $\Gamma \propto Q^{2}$)^23^ models. The authors suggested that the measured dynamics were explained better considering the coupling of the local density fluctuations in the top and bottom leaflets to the bending mode, which corresponds to the second mode of the SL theory.^37^ From their NSE data, they estimated a friction coefficient *b* of the order of 10^8^ Pa s/m.^39^

In 2006, Rheinstädter, Häu*β*ler, and Salditt performed an NSE experiment on highly ordered stacked DMPC bilayers with a significantly improved energy resolution compared to the first studies by Pfeiffer.^65^ They observed two relaxation processes: a faster mode with relaxation time scales between 1 to 10 ns that originated from mixed undulation dynamics and baroclinic modes, and the other slower mode with a relaxation time of about 100 ns originating from the surface relaxation mode.^66^ They estimated the bending modulus $\kappa \approx 14\;k_{B}T$ that reasonably agrees with the values measured with other techniques, and the bilayer surface viscosity was estimated to be 16 times larger than that of water. They also observed softening of the membrane undulations right above the main transition temperature. The anomalous swelling behavior that is known as the inter-bilayer distance is anomalously increased right above the melting transition temperature,^67^ and bilayer softening has been observed at the anomalous swelling regions.^68^ Interestingly, Rheinstädter *et al.* observed membrane softening at a specific length scales of about 420 Å. This length scale is larger than the ripple periodicity, but the authors concluded that the soft mode was linked to the formation of the rippled structure in the lower temperature phase.

Seto and colleagues used non-oriented multilamellar vesicles (MLVs) to observe undulation fluctuations for DMPC bilayers. They applied the ZG model to analyze the NSE data while avoiding the length scale corresponding to the distance between bilayers, where the strong elastic scattering peak hindered the quasi-elastic scattering signal. They observed stiffening of the membranes in the anomalous swelling regimes.^69^ On the other hand, in the work by Kuklin and colleagues,^70^ ULVs were used to measure the relaxation dynamics. In this case, the average relaxation time was faster in the anomalous swelling regions, indicating softening of the bilayers. They concluded that anomalous swelling is induced by the increased short-range repulsive interactions between membranes, and the change in the undulation fluctuations does not affect significantly in the inter-bilayer force balance.

Takeda and colleagues studied free standing lipid bilayers by adding CaCl_2_ to the aqueous solvent to enhance inter-membrane charge interactions, which significantly increases the inter-bilayer distance compared to multi-lamellar stacks.^29,71^ These free standing membranes are an ideal system for the application of the ZG theory in the dynamic range accessed by NSE.^29,71^ Hirai and colleagues measured a mixture of glycosphingolipid, cholesterol (chol), and phospholipid (majority was PC lipid) by means of NSE.^72^ They prepared ULVs by sonicating the lipid aqueous solutions and applied the ZG theory to calculate the bending modulus.

Large unilamellar vesicles (LUVs), which are the current standard membrane geometry for NSE studies of free standing membranes, were first measured with NSE in 2009 by two independent groups.^73,74^ Both groups suggested that the NSE data were reasonably well fit with the ZG model. Arriaga and colleagues examined an application of the theory to the NSE data and concluded it gave a reasonable bending modulus for POPC.^74^ On the other hand, Yi, Nagao, and Bossev measured various saturated and unsaturated PC lipid bilayers and observed an increase in the bending modulus with the bilayer thickness while lipid unsaturation softened the membranes.^73^ These works laid the foundation for the study of undulation dynamics in free standing lipid bilayers using NSE.

In addition, membranes near an air or substrate interface have been studied using grazing incidence neutron spin echo (GINSE). A grazing incident beam can penetrate different depths of the membrane depending on the incident angle, making GINSE a powerful technique to study membrane dynamics as a function of the distance from the interface. The membrane dynamics at interfaces have been studied with GINSE for different lipids and with different additives.^75–79^

### C. Thickness fluctuations

Another class of collective membrane fluctuations that can be measured by NSE is thickness fluctuations. The initial studies of thickness fluctuations were done on soap films, where such fluctuations play an important role during film rupture.^80^ In the 1980s, thickness fluctuations in a solvent free lipid bilayer were reported under equilibrium conditions based on laser light reflection results.^81–83^ Subsequent work by Haskell *et al.* studied thickness fluctuations in thin lipid films using reflectance fluctuation spectroscopy.^84^ Huang estimated the thickness fluctuation amplitude to be several tenths of nanometers for glyceryl monooleate-squalene membranes based on a membrane deformation free energy model.^85^ In MD simulations on the scale of several nanoseconds, spectral decomposition of both undulatory and thickness fluctuations in lipid bilayers was observed.^86^

In nanometer scale surfactant membranes in solution, Farago first realized there were enhanced fluctuations around the length scale of surfactant membrane thicknesses.^31,48^ In an SDS-pentanol–water–dodecane lyotropic phase, he and his colleagues prepared oriented lamellar sheets to measure these dynamics by means of NSE. They found two well-separated relaxation processes: the faster mode was assigned to undulation fluctuations, while the slower relaxations originated from thickness fluctuations, whose relaxation time was estimated to be roughly 3 ns.

On the other hand, Nagao, Seto, and Faraone reported that the membrane undulation dynamics showed a crossover-like step change with *Q* around the length scales of the membrane thickness. They speculated this observation was due to either hydrodynamic and inter-membrane interactions or the influence of the intra-membrane fluctuations on the studied dynamics.^87^ In order to clarify this phenomenon, Nagao designed an experiment to vary the membrane thickness by swelling the surfactant bilayers with oil in C_12_E_5_/octane/water systems.^49^ Together with his colleagues, they showed that the enhanced signal around the membrane thickness originated from the thickness fluctuations by combining NSE experiments and MD simulations.^49,88^ They used the ZG theory to fit the observed ISF, and an excess in dynamics was clearly observed in addition to the undulation fluctuations as

$$\Gamma =\Gamma_{ZG}+\Gamma_{TF},$$ (18)

where Γ_*ZG*_ represents the undulation contributions to the relaxation rate, and Γ_*TF*_ was expressed as a Lorentzian

$$\Gamma_{TF}=\frac{\tau_{TF}^{-1}}{1+{\left(Q-Q_{0}\right)}^{2}\xi^{-2}}\frac{Q^{3}}{Q_{0}^{3}},$$ (19)

where *τ_TF_* indicates the relaxation time of the thickness fluctuations, *Q*_0_ is the peak location of the signature of the thickness fluctuations, and $\xi^{-1}$ is the half width at half maximum of the thickness fluctuation signature. The thickness fluctuation amplitude is calculated as $d_{m}\xi /Q_{0}$, where *d_m_* is the bilayer thickness.

The observed relaxation times for thickness fluctuations in the oil-swollen nonionic surfactant bilayers increased from 5 up to 1000 ns as the bilayer thickness increased.^88^ These experiments were performed using deuterated oil and water, so that the coherent scattering was dominated by the surfactant–surfactant correlations. Nagao further studied the effects of the scattering contrast by replacing deuterated oil with protiated oil and concluded that the thickness fluctuations could be measured in both scattering contrasts, but the use of deuterated oil was best for more detailed measurements of the thickness fluctuations.^89^

Woodka and colleagues were the first to measure thickness fluctuations in lipid LUVs using NSE.^41^ Keeping the same headgroup chemistry, the effects of lipid tail length on the thickness fluctuations signals were studied by employing acyl tail-deuterated DMPC-d54, DPPC-d62, or DSPC-d70 ULVs in D_2_O. The ISF measured by NSE followed the ZG prediction; however, a clear enhancement in the dynamics was observed around the length scale of the bilayer thickness. The relaxation time of the fluctuations was estimated to be on the order of 100 ns, which was much slower than the oil-swollen surfactant bilayers with a similar bilayer thickness,^49^ and the fluctuation amplitude was approximately 10%–20% of the bilayer thickness in the fluid phase. In the gel phase, no such enhancement was observed due to much slower dynamics, which was consistent with the computer simulation result by West and Schmidt.^90^

It is noteworthy that the thickness fluctuation amplitude can be related to the area compressibility modulus of the membrane. The area compressibility modulus is^86^

$$K_{A}=\frac{k_{B}T}{\sigma_{A}^{2}A_{L}},$$ (20)

where *σ_A_* is the fractional change in area, and *A_L_* is the area per lipid. Assuming conservation of the molecular volume of the lipids, then $\sigma_{A}=\delta A/A=\delta L/L$, where *A* and *L* are the molecular area and length, respectively. The relative change in the width of the peak assigned to the membrane thickness fluctuations corresponds to the thickness fluctuation amplitude, $\delta L/L=\xi /Q_{0}$. The thickness fluctuation amplitude is thus written using *K_A_* and *A_L_* as^1,42^

$$\frac{\xi }{Q_{0}}=\sqrt{\frac{k_{B}T}{K_{A}A_{L}}}.$$ (21)

Meanwhile, BSO theory predicted the relaxation time of the thickness fluctuations, *τ_TF_*, and depended both on *K_A_* and the solvent and membrane viscosities *η* and *η_m_* as^50^

$$\tau_{TF}^{-1}=\frac{kK_{A}}{\eta_{m}k+2\eta }\approx \frac{K_{A}}{\eta_{m}},$$ (22)

where *k* is the wavenumber of the fluctuations. This approximation applies when the wavelengths are shorter than the Saffman–Delbrück length, $l_{SD}=\eta_{m}/\eta$, which is the case for the lipid bilayers.^50^ Bradbury and Nagao applied this relationship to an oil-swollen surfactant membrane composed of C_12_E_5_/SDS/decane/water and estimated membrane viscosity from the NSE measurements of the thickness fluctuations.^91^ Notably, the estimated value of the membrane viscosity from the NSE data was consistent with the values from rheological measurements of the mixtures of C_12_E_5_, SDS, and decane.^91^

Another commonly used relationship for *κ* and *K_A_* from the thin sheet theory is^92^

$$K_{A}=\frac{\beta \kappa }{d_{m}^{2}},$$ (23)

where *β* represents a coupling constant between *K_A_* and *κ*. The value of *β* varies depending on the interleaflet coupling of the bilayer, where smaller values correspond to more coupled leaflets.^92,93^ Thus, combining Eqs. (21) and (23)

$$\beta =\frac{d_{m}^{2}Q_{0}^{2}}{A_{L}\xi^{2}}\frac{k_{B}T}{\kappa },$$ (24)

and the observed thickness fluctuation relaxation times can be used to calculate the membrane viscosity^42^

$$\eta_{m}=\frac{\beta \kappa }{d_{m}^{2}}\tau_{TF}=\frac{k_{B}TQ_{0}^{2}}{A_{L}\xi^{2}}\tau_{TF}.$$ (25)

Thus, one can estimate the coupling constant *β* as well as the membrane viscosity *η_m_* by combining SANS and NSE data. A value of *β* = 24 is predicted from the polymer brush theory for typical lipid bilayers.^93^ The first application of this analysis approach to NSE data for DMPC, DPPC, and DSPC bilayers assuming *β* = 24 gave *η_m_* values of the order of 10 nPa s m,^42^ which were slightly larger than the estimates by other techniques but within the range of broadly distributed *η_m_* values in the literature.^94^ It is also important to note that the value of *β* can change when different types of molecules are added to the membrane and affect the interleaflet coupling. Therefore, NSE data can be used to estimate four important membrane parameters, namely, the bending modulus *κ*, the area compressibility modulus *K_A_*, the membrane viscosity *η_m_*, and the coupling constant *β*. In Secs. III D–III I, we review the effects of various additives on these properties that have been studied with NSE.

### D. Effects of mixing lipids on the membrane fluctuations

In order to know the effects of hydrocarbon tail length on the membrane dynamics, an equimolar mixture of DMPC and DSPC was measured with NSE, in which Ashkar *et al.* showed that both undulation and thickness fluctuations were enhanced compared to a pure DMPC or DSPC bilayer.^95^ Kelley *et al.* further studied these mixtures by systematically changing the molar ratio between DMPC and DSPC.^96^ The mixed bilayers were thinner than the composition-weighted average expected based on the single component membranes, while the lipid volumes followed predictions assuming ideal mixing. This suggested that the lipid packing within the bilayer was disrupted and *A_L_* increased in the mixtures. Because of such disruption of the molecular arrangements, the membrane became more dynamic. Interestingly, these changes in the dynamic parameters were well explained by the variation in *A_L_*. Each dynamic parameter, *κ*, *K_A_*, and *η_m_*, followed their respective scaling functions with *A_L_* that were predicted in some theories for membrane dynamics. The result emphasized the synergy between structure and dynamics of the lipid bilayers.^96^

Brüning and colleagues studied undulation fluctuations of DMPC upon addition of lipids with unsaturated acyl tails or charged head groups.^97^ For this study, DMPC was replaced with either DOPC (monounsaturation in both acyl tails) or DOTAP (monounsaturation in both acyl tails and cationic head group) up to a mole fraction of 50% (50 mol. %). The bending modulus *κ* decreased as the amount of DOPC was increased. A similar reduction of *κ* was observed for the mixtures of DMPC and DOTAP, and the authors concluded that the charge effects were much less significant than the effects of the unsaturated acyl tails.

Cholesterol (chol) is an essential lipid species in eukaryotic cells, and as such, the effects of chol on the membrane structure and dynamics are still actively studied. In a study by Hirai and colleagues,^72^ a decrease in *κ* was seen when glycosphingolipid was mixed to DOPC or POPC at a molar fraction of 10%, while addition of chol made the membrane more rigid in the fluid phase. The authors pointed out that the ternary mixture of glycosphingolipid, PC, and chol had a low value *κ*, suggesting the formation of raft domains softened the membrane and made it more fluid. Arriaga and colleagues measured effects of chol on POPC bilayers.^98^ Considering the contribution from translational diffusion of the vesicles in the analysis, they observed about twofold increase in the bending modulus as the chol increased up to 50 mol. %. They concluded that the observed stiffening effect of chol on POPC bilayers was consistent with the structural condensation caused by hydrogen-bonding between the phospholipids and cholesterol. On the other hand, when Brüning and colleagues measured fluctuation dynamics of DMPC with chol bilayers, they observed a much larger increase in the DMPC membrane bending rigidity. The sixfold increase in the bending modulus of DMPC membranes containing 40 mol. % chol suggested that the POPC molecules partially diminished the condensing effect of chol due to mono-unsaturation in a single acyl tail.^99^

Arriaga and colleagues further analyzed the dynamics in POPC/chol mixed bilayers considering the hybrid compression-curvature modes and found that the intermonolayer friction coefficient, *b*, increased with increasing chol concentration,^43^ which was consistent with a fluorescent spectroscopy study.^100^ The results suggested chol increases the coupling between POPC leaflets and that chol can act as a coupling agent between leaflets. Furthermore, they suggested that chol makes lipid membranes stiffer but also more fluid as it increases the friction between the leaflets.^101^ These results, the increase in *κ* for DMPC and POPC bilayers observed by NSE, are consistent with the previous observations with other techniques.^102,103^

Chakraborty and colleagues used NSE, solid state deuterium NMR, and MD simulation and observed an increase in the bending modulus with increasing chol concentration in DOPC bilayers.^104,105^ They concluded that chol locally increases the bending rigidity of DOPC membranes due to an increase in the packing order. However, previous studies of the same lipid mixtures using micropipette aspiration,^106^ XDS,^103^ shape fluctuation spectroscopy, and electrodeformation measurements^107^ suggested that chol had almost no effect on the bending modulus of mixed DOPC and chol membranes. These controversial results led to several discussions in the field.^108,109^ An interesting point raised by Nagle *et al.*^108^ is that the techniques used in Chakraborty *et al.* measured the bending dynamics in the time domain and were therefore sensitive to various internal membrane dissipation mechanisms, while other more commonly used static techniques or long length scale measurements are insensitive to these effects.^110^ This point is still an ongoing discussion but suggests NSE can provide new insight into the role of cholesterol on the dynamics at short length and time scales because NSE is sensitive to the internal dissipation in the membrane.

Mixtures of different lipid molecules are also biologically relevant. For example, myelin membranes are composed of a variety of different molecules, and the myelin sheath is a multilamellar membrane wrapped around axons of neurons to insulate. Multiple sclerosis is a neurodegenerative disease leading to demyelination and axon damage in the human central nervous system. Model mimics of the native and diseased membrane compositions are well-characterized, and Krugmann and colleagues studied structure and dynamics of such mimic myelin membranes. They prepared ULVs with native and diseased membrane mimics and observed an approximate 25% reduction of the lipid membrane bending modulus of the diseased mimic. Although the diseased mimic membranes contained more cholesterol, the amount of sphingomyelin also decreased, and the decrease in *κ* was attributed to the decrease in sphingomyelin content in the membrane.^111^

### E. Effects of peptides and proteins on bilayer fluctuations

In order to establish how the elastic properties of soft membranes are affected by protein–membrane interactions, a variety of NSE studies have been performed on lipid bilayers containing various peptides or proteins. Lee and colleagues started this line of studies by incorporating a pore-forming antimicrobial peptides, melittin, into DOPC bilayers.^40^ At low melittin concentrations, below which the ULVs were not all perforated, the bending modulus of the DOPC bilayers decreased with added peptide as the melittin perturbed the acyl tail packing. As the melittin concentration, and the corresponding density of membrane spanning pores increased, the value of *κ* also increased. This increase was likely due to the high modulus of the pores and inter-pore interactions which had a stiffening effects on the DOPC bilayers.

The effects of similar pore-forming antimicrobial peptides, gramicidin and alamethicin, on saturated DMPC bilayers were studied by Kelley and colleagues.^112^ At low concentrations where the added peptides had no effect on the overall membrane structure, the effective rigidity of the membranes changed by up to a factor of 2, with gramicidin stiffening the membrane and alamethicin softening the membrane. They also observed enhanced thickness fluctuations at low gramicidin concentrations, while the thickness fluctuations were dampened all studied alamethicin concentrations as well as at higher concentrations of gramicidin. They suggested that the incorporation of gramicidin at low concentrations accelerated the membrane dynamics by decreasing the membrane viscosity, and these enhanced fluctuations that may help increase the probability of gramicidin dimerization needed to form transmembrane pores as was theoretically predicted in work by Helfrich and Jakobsson.^113^ Their results suggested that adding peptides may simultaneously increase the bending modulus while decreasing the membrane viscosity, which may indicate that the elasticity and viscosity of membranes can be tuned independently. Moreover, these studies highlight that the general term of “fluidity” does not always correspond to changes in membrane elasticity.

In a slightly more complicated bilayer mixtures, effects of amyloid-*β* proteins on the structure and dynamics were studied by Hirai and colleagues.^114^ They prepared LUVs composed of a glycosphingolipid (G_*M*1_), chol, and DOPC or DPPC. Complementary SAXS studies showed that the G_*M*1_-chol domains were preferentially located on the outer leaflets. In turn, amyloid-*β* proteins are assumed to be bound to the negatively charged G_*M*1_-chol rich domains. The NSE studies showed that the fluid lipid membranes were significantly stiffened due to the amyloid-*β* binding to the G_*M*1_/chol/DOPC membranes, while only a slight increase in the bending modulus was measured for the gel phase membranes (G_*M*1_/chol/DPPC). Based on these findings, the authors concluded that such a change in membrane dynamics could be involved in the onset of some amyloid disorders diseases. Moreover, Heller and Zolnierczuk showed that the conformational change of a bound HIV-1 fusion peptide derivative increased the bending modulus of DMPC/DMPG (a molar ratio of 7/3) LUVs.^115^ Circular dichroism spectroscopy indicated that peptide had an *α*-helical conformation at a low concentrations (peptide to lipid ratio, P/L≈1/200) but adopted a *β*-sheet conformation at a high peptide concentration ( P/L≈1/50). The bilayers were also thickened and stiffened by addition of these high peptide concentrations. As the *β*-sheet conformation is related to fusion in the native fusion protein sequence, the observed increase in bending modulus could have implications for the function of the fusion peptide.

Compiling the results of these studies suggests that the addition of peptides that form *β*-sheet tend to stiffen lipid bilayers, while the effects of *α*-helical peptides appear to be more complicated. To better understand the effects of *α*-helical peptides on the membrane structure and dynamics, Qiang and Zolnierczuk studied the effects of aurein 1.2 peptide, one of the shortest *α*-helical antimicrobial peptide found in nature, on a model lipid membrane composed of DMPC and DMPG using a combination of SANS and NSE.^116^ Upon addition of the peptide, the membrane became softer at P/L=1/100, while at a higher peptide concentration of P/L=1/30, the membrane became stiffer. The authors suggested that the changes in the membrane properties caused by the peptides are due to a delicate balance of several competing factors. A detailed understanding of these different factors is imperative to precisely control or modulate the interactions when improving the performance of antimicrobial peptides. For example, recent studies of the effects of SARS-CoV-2 fusion peptide on the properties of biomembranes showed incorporation of the peptides made the membrane more rigid.^117^

Instead of encapsulating peptides or proteins into membranes, Gilbert and colleagues encapsulated two different proteins, *β*-galactosidase or aspartic protease, into the water channels in lipid sponge phase nanoparticles.^118^ These enzymes are used in various processes in the food industry. Combined studies of the protein and membrane properties showed that enzymes retained their activity in the water channels and also stiffened the host membrane composed of diglycerol monooleate, glycerol monooleate, and polysorbate that encapsulated the water channels. The stiffening effects seen in the case of aspartic protease were most likely caused by hydrogen bonding between amino acids in the protein and carbonyl groups in the lipid head groups, which replaced the water–carbonyl hydrogen bonds, as shown by the MD simulations.

### F. Effects of small molecule drugs on membrane fluctuations

Nonsteroidal anti-inflammatory drugs (NSAIDs) are the most widely used drugs for their antipyretic, analgesic, and anti-inflammatory actions. The effects of the NSAIDs on the membrane dynamics have been studied using NSE by several groups. Boggara and colleagues studied effects of pH and ibuprofen on the dynamics of DMPC bilayers.^119^ The membrane rigidity was almost constant between pH = 5 and 7, but lower at low pH. Upon addition of ibuprofen, the bilayers became softer, and the rigidity did not change significantly with changing pH. By combining SANS and MD simulations, they explained the results considering the lipid head group hydration and ibuprofen partitioning into the membrane. Contrary to this result, Jaksch and colleagues observed a stiffening effect in GINSE studies of SoyPC bilayers containing ibuprofen.^76^ They suggested this stiffening might be related to long-term toxic effects of some NSAIDs. Sharma and colleagues studied general effects of NSAIDs, including ibuprofen, aspirin, and indomethacin, on the dynamics of DMPC bilayers.^120^ Their NSE results showed a softening of the bilayers upon addition of the three NSAIDs. They concluded that incorporation of NSAIDs modulates the mechanical properties of the membrane, which may in turn affect physiological processes. The effects of acetaminophen on the DOPC bilayers were studied by De Mel and colleagues.^121^ Although acetaminophen is not considered an NSAIDs because it does not have significant anti-inflammatory activity, like NSAIDs, acetaminophen is a small aromatic molecule used to treat pain. De Mel *et al.* observed softening of the membrane upon addition of acetaminophen using NSE, but SANS suggested the bilayer structure was unchanged. They further suggested that the short time lipid motions were enhanced by the addition of acetaminophen because the drug molecule increased the space between lipid molecules.

The effects of a local anesthesia, lidocaine, on membrane dynamics have also been studied with NSE. Yi and colleagues demonstrated that lidocaine modified the bilayer structure by increasing the *A_L_* and decreasing the thickness of DMPC bilayers containing with 5% of DMPG.^122^ The NSE measurements showed an increase in the bending modulus in the fluid phase upon lidocaine addition, while the temperature dependent change of the bending modulus was less steep across the main transition temperature. Similar effects were observed when a saponin, aescin, was incorporated into DMPC bilayers.^123^ Aescin had anti-inflammatory and anti-edematous properties and is used to treat chronic venous insufficiency. They observed softening of the membrane below the main transition temperature of DMPC, while the membrane becomes more rigid at high temperatures with aescin addition. They concluded that the large triterpenic backbone stiffened the bilayers at high temperatures, while hydrogen bond formation between the hydroxyl groups of aescins sugar moieties with the carbonyl and negatively charged phosphate groups of DMPC seems to have an important effect on the bending modulus at low temperatures.

The effects of vitamin E acetate, which is a potential instigator of electronic-cigarette/vaping-associated lung injury (EVALI), on the dynamics of a model pulmonary surfactant without proteins have also been studied with NSE.^124^ DiPasquale and colleagues constructed pulmonary surfactant mimics composed of DPPC, POPC, POPG, and chol and investigated the effects of varying amounts of vitamin E acetate on the membrane rigidity. Interestingly, the pulmonary mimic was more rigid than the pure DPPC bilayers; however, the addition of vitamin E acetate softened both the pulmonary surfactant mimic and pure DPPC bilayers. The authors stated that although it remains to be seen if vitamin E acetate is the sole culprit of EVALI, the data suggest vitamin E acetate is capable of reducing the elastic properties of the pulmonary surfactant that plays a vital role in respiration.

Cardiolipin (CL) lipids are a class of anionic phospholipids that are found predominantly in the inner mitochondrial membrane of eukaryotic cells and in the plasma membranes of certain bacteria. This class of lipids is composed of two phosphate moieties, and the linkage between the two phosphates restricts their mobility and reduces the headgroup volume compared to other classes of phospholipids. This smaller headgroup volume is what allows CL lipids to form non-lamellar structures. Pan and colleagues studied the structure and dynamics of tetraoleoyl CL bilayers using densitometry, SANS, SAXS, and NSE together with MD simulations.^125^ They measured the molecular volume, area per lipid, and the bilayer thickness together with the bilayer bending modulus. Notably, the results showed that CL bilayers had a much larger *κ* than DMPC or DOPC bilayers despite also having a significantly larger *A_L_*. Castillo and colleagues mixed a model CL with other lipids to create a more complex mimic of mitochondrial membranes and studied the effects pancratistatin, a natural anti-cancer agent, on the dynamics of the inner mitochondrial membrane.^126^ They observed an increase in the bending modulus upon addition of pancratistatin, and the changes in membrane properties could result in the relocation and release of cytochrome c to initiate the apoptosis cascade in cells.

### G. Effects of other additives on membrane fluctuations

There are a number of other organic and inorganic compounds found in nature that interact with biological membranes. Inspired by compounds that are known to contaminate the food chain, Brüning and Farago studied the effects of perfluorooctanoic acid on the mechanical properties of DMPC bilayers.^127^ Perfluorinated compounds are widely used to prepare fire-extinguishing foams, anticorrosion agents, and lubricants and are known to persist in the environment.^128^ As such, Brüning and Farago measured the NSE signal from the DMPC ULVs with and without a mole fraction of 5% or 10% perfluorooctanoic acid, and the membrane relaxation dynamics were observed to slow down. This slowdown was associated with an increase in the bending modulus of the DMPC bilayers upon addition of perfluorooctanoic acid.

Usuda and colleagues studied effects of alkanes on the dynamics of DPPC bilayers.^129^ They kept the mole fraction of alkane constant 40% and increased the chain length *i* from octane (C8) to tetradecane (C14). The longer alkanes were observed to condense in the bilayer center. Although the bilayer thickness did not increase significantly, *κ* decreased while the *K_A_* values extracted from measurements of the thickness fluctuations increased with increasing the number of carbons in the alkanes. The opposite trends in *κ* and *K_A_* were explained as a change in *β*, and the authors suggested that intermonolayer coupling decreased when the oil molecules condensed at the center of the bilayers. Misuraca and colleagues studied the effects of linear and branched alkanes on the membrane dynamics in model protocell membrane vesicles.^130^ The effects of the studied alkanes depended on the temperature, where softer membranes were formed at low temperatures, while slightly more rigid bilayers were formed at high temperatures. As the membrane stability increased at high temperatures upon addition of the alkanes, the authors concluded that the alkanes helped maintain the lamellar structure of the protocell membranes at high temperatures, as these lipid compositions would prefer to form micelles at these temperatures if the alkanes were not incorporated. As such, the inclusion of alkanes into protocell membranes might have been one way early cells survived at high temperature conditions.

Instead of specifically incorporating hydrocarbon chains in the hydrophobic region of the lipid bilayers, Kumari and colleagues incorporated an imidazolium ionic liquid to DMPC bilayers.^131^ The ionic liquids were dispersed at the bilayer–water interface, and the NSE results indicated the bending modulus increased when the hydrophobic cation was incorporated into the bilayer. Hoffmann and colleagues studied the influence of the adsorption of small silica nanoparticles to the outer vesicle surface.^132^ While the silica nanoparticles did not significantly affect the vesicle structure, they did make the membrane softer. Meanwhile, Chakraborty and colleagues studied the effects of encapsulating hydrophobically modified gold nanoparticles into the hydrophobic region of the bilayers on the membrane dynamics.^133^ They observed a softening of DPPC bilayers upon incorporation of the gold nanoparticles.

Another class of additives that interacts with lipid bilayers is polymers. Polymers are sometimes used as to coat liposomes for drug delivery applications to introduce “stealth” properties^134^ or used to protect cell membranes from various dysfunctions. To understand how adding polymers affect the membrane structure and dynamics, De Mel and colleagues mixed poly(ethylene oxide)-mono-n-octadecyl ether with DOPC bilayers.^135^ The hydrophobic octadecyl group attached to the polymer resided in the lipophilic hydrocarbon core of the bilayer and disrupted the lipid packing, and thereby lead to a reduction in the membrane bending modulus. Contrary to this result, Wang *et al.*^136^ and Yu *et al.*^137^ showed that incorporation of triblock copolymers stiffened bilayers. Wang and colleagues prepared poly(methacryloyloxyethyl phosphorylcholine)-poly(propylene oxide)-poly(methacryloyloxyethyl phosphorylcholine) triblock copolymers that effectively function as membrane-targeting cellular rescue agents.^136^ The bending modulus of DMPC bilayers increased upon addition of the block copolymer both in the fluid and gel phases. Likewise, Yu and colleagues studied the effects of an end-phosphorylated polyethylene glycol triblock copolymer with a hydrophobic bisphenol A center on the membrane properties.^137^ This class of polymer is used as the virulence-directed agent for treating diseases and disorders involving microbial pathogens. Although the polymer did not change the membrane structure significantly, an increase in the membrane bending modulus was observed. These studies highlight the importance of the end functional groups in determining the effects polymers have on lipid vesicles. Studies on the effects of polymers on membrane dynamics should be extended to understand the effects of extracellular macromolecules found in nature in the future.

### H. Effects of domains and asymmetry on membrane undulation fluctuations

Here, we see how internal membrane structures affect the membrane dynamics. The first example is the bending modulus measured in phase separated DMPC and DSPC mixed bilayers with coexisting fluid and gel domains.^138^ As DMPC and DSPC differ in acyl tail length by four carbons, the melting transition temperature differs about 30 K. Depending on the mixing ratio between DMPC and DSPC, the membrane segregates into gel and fluid phases in-between the melting transition temperatures and forms global domains. Kelley, Butler, and Nagao measured the bending fluctuations in this gel–fluid coexistence region and showed that the effective bending modulus followed the area fraction of the gel domains. Furthermore, the experimental data were well explained using a theoretical model for the effective rigidity of a membrane containing an inhomogeneous distribution of a rigid phase.^139^ On the other hand, studies by Hirai and colleagues focused the dynamics of membranes containing small domains, specifically, model raft-domains, to study the overall membrane properties.^72,114^

A completely different approach was taken by Nickels and colleagues, in which they focused on the dynamics of small raft domains by employing the neutron contrast matching technique.^140^ They isolated the ≈13 nm diameter nanodomains residing in ≈60 nm ULVs by employing appropriate deuteration schemes. The observed bending modulus of the nanometer sized domains appeared to be different from the modulus of the surrounding continuous phase. The results suggested that mismatches in bending modulus between domains and the matrix should be accounted for when explaining the emergence of lateral heterogeneities in lipid systems. On the other hand, lateral diffusion of nanometer sized domains on lipid vesicles was studied by Sakuma and colleagues using NSE.^141^ The obtained diffusion coefficient was explained in terms of the hydrodynamic model of Brownian objects in a fluid membrane.^142^ These examples lay the foundation for the studies of the dynamics of domains in lipid bilayers, which we expect will further be explored using neutron scattering techniques in future.

In contrast to the effects of lateral heterogeneity in membrane caused by the presence of domains, Rickeard and colleagues considered the effects of transverse heterogeneity in membrane properties by studying the dynamics of asymmetric bilayers.^143^ The asymmetric vesicles were composed of an outer leaflet enriched in egg sphingomyelin (ESM) and an inner leaflet enriched in POPE and prepared using a cyclodextrin-mediated exchange protocol. Notably, the bending fluctuations of the asymmetric bilayers measured by NSE showed trends not predicted by their symmetric counterparts. The asymmetric bilayers show suppressed bending fluctuations compared to the symmetric bilayers, and the authors concluded that the compositional asymmetry and leaflet coupling influence the internal dissipation within the bilayer and result in membrane properties that cannot be directly predicted from corresponding symmetric bilayers.^143^

Since biomembranes are much more complicated than synthetic model membranes, the bottom up approach taken in the examples shown in this subsection help develop a fundamental understanding of the properties of membranes that will be useful in future studies of more biologically relevant systems.

### I. Undulation fluctuations in more biologically relevant membranes

In this subsection, we overview NSE studies more complex biologically and relevant systems, cell-derived membranes, and even cells themselves. Nickels and colleagues studied the fluctuation dynamics of *Bacillus subtilis* lipid extract, which contained PE, PG, CL, and lysyl-PG lipids in addition to neutral lipids (mostly diacylglycerol).^144^ The extract also contained a distribution of saturated branched and unbranched fatty acids. They then measured the bending modulus of the lipid vesicles prepared from the lipid extract and compared the results with molecular dynamics simulations. More recently, Himbert and colleagues measured human red blood cell membrane dynamics using a combination of XDS, NSE, and computer simulations.^145^ All these techniques showed that the red blood cell membranes without cytoskeletal networks are softer than synthetic model biological membranes. The authors suggested that nature has designed the red blood cell to be soft for reasons including in the permeability of gas molecules and to accommodate possible local area changes.

*In situ* studies of photosynthetic machinery in cyanobacterial cells were performed by Stingaciu and colleagues.^146,147^ The flattened membrane structure in cyanobacterial cells, called thylakoids, responds to variations in environmental conditions, and there are corresponding changes in the membrane properties. In particular, the thylakoid membrane undulatory motions *in vivo* were measured with and without light exposure, and the authors observed that the membranes were softer in the dark conditions than in high light conditions. They concluded that electron transfer between photosynthetic reaction centers and the associated electrochemical proton gradient across the thylakoid membrane result in a significant driving force for excess membrane dynamics.^146^ They further studied the effects of disrupting the photosynthetic electron transfer by treating the thylakoid membrane with a chemical: 3-(3,4-dichlorophenyl)-1,1-dimethylurea. They found that the disruption pathway rigidified the native membranes in the dark cycle yet softened the membrane in light conditions. A slowdown of the membrane fluctuations under the dark condition was also observed for thickness fluctuations. The disrupted electron transfer chain and the decreased proton motive force within the lumenal space partially explain the observed changes in the mechanical properties of the membranes and support the hypothesis that the photosynthetic process is tied to thylakoid rigidity in the cyanobacterial cell.^147^

## IV. MOLECULAR MOTIONS IN LIPID MEMBRANES

The collective membrane fluctuations discussed in Sec. III are limited to length scales of the long and short wavelength cutoffs. The long wavelength cutoff is usually taken as the particle or membrane patch sizes, while the short wavelength cutoffs corresponds to approximately the membrane thickness. The dynamics of lipid and surrounding molecules at even shorter length scales and close to the molecular scales have also been investigated by means of incoherent QENS.^5,6,10,148^

A comprehensive series of experiments on lipid molecular motions were performed by Sackmann and co-workers starting in 1988. Pioneering work using an ultracold neutron gravity spectrometer was performed by Pfeiffer *et al.*, and they determined that the lateral diffusion constant of a lipid molecule was $D=2.6\times 10^{-7}{\text{cm}}^{2}/s$ which was slightly larger than the value obtained by the photobleaching technique.^149^ They then extended their study to verify the details of dynamical behavior of lipid molecules by incoherent QENS. They showed the differences in the dynamics of individual lipid molecules in the gel and fluid phases and interpreted their properties applying the concept of the packing density.^150,151^ They also investigated the dynamics of hydration water using perdeuterated DPPC (DPPC-d75) deposited on a substrate and solvated with either H_2_O or D_2_O. They observed no anisotropy of the water dynamics normal and parallel to the lipid membrane depending on the orientation. Moreover, only rotational motions of water molecules were observed at low hydration levels, and jump diffusive motions similar to bulk water were seen in highly hydrated membranes.^152^ In the final paper by these authors, they investigated the hydration dependence of the local diffusion as well as the acyl tail motions of DPPC molecules. The results showed that the QENS spectra from the fluid phase were not explained by acyl tail dynamics alone, and the diffusion of the molecule within its solvation cage must also be considered to fit the data.^153^

In 2005, Rheinstädter *et al.* published their first paper on the dynamics of lipid molecules and the hydration water.^154^ They measured the dynamics of a mixture of DMPC-d54 and D_2_O with a BS spectrometer and observed coherent scattering from the acyl tails and interstitial water. Based on these results, they discussed the freezing of the acyl tails and reported a second freezing transition that they attributed to the hydration water. In the following papers, they identified two types of motions; a fast relaxation process associated with the translational diffusion of the lipid and water molecules, and the slow collective motion in lipid membranes occurs over several lipid distances.^19,155,156^ Following these works, Busch *et al.* investigated the mechanism of long-range diffusion in DMPC lipid membranes using a TOF spectrometer.^157^ They concluded that the lipid dynamics were not confined in the fluid phase, and individual lipid molecules flow with their neighbors as a dynamically assembling patch across the membrane.^157^ Meanwhile, Wanderlingh *et al.* measured QENS spectra for POPC and DOPC and showed the presence of three types of motions: slow diffusion of the entire phospholipid molecules in a confined cylindrical region, conformational motions of the acyl tails, and fast uniaxial rotations of the hydrogen atoms around their carbon atoms.^158^ The most recent developments on the local dynamics of lipids were made by Peters and co-workers. They built a theoretical framework and derived the corresponding analytical expressions for the ISFs measured with QENS. The model is named “dynamical Matryoshka model” and describes the dynamics of lipid molecules as a nested hierarchical convolution of three motional processes. This model was then used to fit the QENS signals from DMPC and DMPG membranes and successfully described the dynamics in supported bilayers as well as MLVs.^159–161^

The effect of additives on the lipid molecular motions have also attracted the attention of several research groups. Rheinstädter and co-workers studied the effect of chol on the nanosecond lipid dynamics using triple-axis, BS, and NSE spectrometers. They found that adding chol resulted in a pronounced stiffening of the membranes on the nanometer length scale both in the gel and fluid phases, and at the same time, the collective nanoscale diffusion was significantly slower.^162–164^ Sharma *et al.* investigated the effect of antimicrobial peptides on the dynamics of DMPC membrane and showed that the peptides caused an enhancement in the lateral motions of lipid molecules in the gel phase but had a stiffening effect in the lipid fluid phase. Additionally, they showed the effects of the peptides on the lipid dynamics disappeared in the membranes containing 20 mol. % chol.^165,166^ Sharma *et al.* also investigated the effects of an amyloid *β* peptide as well as aspirin on the membrane dynamics and discussed the potential relationships between the effects of these added molecules and their biological functions.^120,167^ They further extended their studies to reveal the dynamics of a membranes made from liver lipid extracts in the presence of an ionic liquid.^168^ A recent contribution by Santamaria *et al.* focused on how the SARS-CoV-2 fusion peptide affected the membrane properties and the influence of Ca ions and chol in the fusion process. From a set of NR, SANS, and QENS experiments, they proposed a molecular mechanism for SARS-CoV-2 cell entry.^117^

Following the pioneering work by König *et al.* in 1994, the hydration water dynamic in the vicinity of lipid membranes was revisited by Swenson *et al.* in 2008.^169^ They prepared three samples: fully protiated DMPC with heavy water (DMPC/D_2_O), acyl tail deuterated DMPC with heavy water (DMPC-d54/D_2_O), and acyl tail deuterated DMPC with light water (DMPC-d54/H_2_O). The dynamical behavior of the water molecules was obtained by subtracting the QENS data measured for the DMPC-d54/D_2_O sample from that of DMPC-d54/H_2_O. From the *Q* dependence of the relaxation time, they concluded that the water relaxation process is that of jump diffusion, and the diffusion constant was lower than that of bulk water by only a factor of two.

In 2015, further QENS experiments on the dynamics of hydration water molecules in orientated membranes were performed by Rheinstädter and colleagues.^170,171^ They analyzed the QENS data using the Kohlrausch–Williams–Watts function and concluded that the hydration water dynamics were anisotropic and sub-diffusive in nature at nanometer-length scales. In order to compare the dynamics of water and lipid molecules, Yamada *et al.* prepared two samples: perdeuterated DMPC (DMPC-d67) with H_2_O and protiated DMPC with D_2_O.^172^ They categorized the hydration water into three types: free water with dynamics slightly different from that of bulk water, loosely bound water with dynamics one-order of magnitude slower that of free water, and tightly bound water with comparable dynamics to that of DMPC molecules. The slow dynamics of the loosely and tightly bound water compared to bulk water were also reported in a MD simulation, where the tightly bound water that form strong hydrogen bonds with DMPC had a translational diffusion coefficient 20 times smaller than that of bulk water.^173^ These QENS and MD simulation results also were quantitatively consistent with those measured by DSC^174^ and terahertz spectroscopy.^175^ Further studies have been done to investigate the effects of metal cations bound at phospholipid headgroups on the hydration water dynamics.^176^ The results suggested that the number of loosely bound water molecules is determined by the nature of the phospholipid membrane.

## V. COLLECTIVE DYNAMICS OF ACYL TAILS

So far, we have seen studies of collective membrane dynamics in which NSE was used to measure the undulation and thickness fluctuations on length scales similar to or greater than the bilayer thickness. At the molecular level, measuring the incoherent scattering originating from hydrogen atoms in lipid or water molecules using BS or TOF instruments, molecular motions such as diffusion of the individual lipid and water molecules as well as acyl tail motions were studied. In addition to these studies, collective motions of the acyl tail structural correlations have been measured to further understand the origins of membrane properties. The acyl tail correlation peak seen in scattering data at Q≈1.5 Å^−1^ originates from the lipid molecular packing and has traditionally measured in x-ray diffraction to determine the structure of the lipid molecular packing and to estimate the distances between the acyl tails and/or *A_L_*.^177–180^ These correlation peaks are also observable in neutron scattering measurements when the hydrogens in acyl tails are replaced with deuterium.

Chen and colleagues were the first to measure the collective acyl tail correlation dynamics in DLPC membranes using inelastic x-ray scattering.^181^ They observed three eigen modes, two of which appeared at the energy scales of ≈±7 meV (corresponding to sub ps time scales) and the other appeared around an energy exchange ω≈0. They concluded that the two of the three eigen modes originated from the sound mode, and the other mode was related to the thermal diffusivity of the lipids. Rheinstädter and colleagues built upon this study and measured INS from employing DMPC-d54 membranes.^182^ They also observed the three eigen modes, and in addition, they observed an additional mode at ω≈0 which they attributed to additional intramolecular degrees of freedom and contributions from incoherent scattering.

Further neutron spectroscopy studies by Rheinstädter and colleagues using a higher energy resolution BS spectrometer allowed them to probe energy ranges from ∼1 to ∼10 μ eV.^156^ They measured the dynamics of DMPC in D_2_O, which were predominantly the incoherent dynamics from the hydrogen atoms in the DMPC molecules. They also measured the dynamics of DMPC-d54 in D_2_O, which were predominantly the coherent dynamics of the acyl tail correlations. Comparing the spectra from the two samples showed that contrary to the incoherent dynamics, where the relaxation time decreased with increasing *Q*, coherent signal showed a maximum relaxation time of about 50 ps in the fluid phase at *Q* values corresponding to the acyl tail correlation peak.

Nagao and colleagues extended the energy resolution further by employing NSE and Mössbauer time domain interferometry (MTDI)^183^ and study the acyl tail correlation dynamics over a combined dynamic range from a few ps up to 300 ns.^184^ In the fluid phase of DMPC bilayers, they observed two relaxation modes with relaxation times of ≈30 and ≈500 ps, and the relaxation times did not change significantly with temperature. The relaxation time of the faster mode measured in these studies was consistent with the previous observation by Rheinstädter *et al.*,^156^ while the slower mode measured with NSE was outside of the energy resolution of the BS spectrometer and not seen in the previous studies. Nagao *et al.* explained these two modes as the density modes of the acyl tails (faster mode) and of the lipid molecules (slower mode). Interestingly, the density mode of the acyl tails was about an order of magnitude slower than the structural correlation dynamics of an analogous linear alkane (tetradecane) that has the structural relaxation times of about 3 ps in a bulk solution.^185^ On the other hand, the slower mode was attributed to rearrangements of the lipid molecules within the membranes, and the measured relaxation time agreed well with a previous NMR study of lipid diffusion.^186^ In the gel phase, the structural correlations were heterogeneous, and the measured relaxation time changed with varying temperatures from 10 to 100 ns.

Employing a relationship for the structural relaxation time scales and the macroscopic liquid viscosity in three dimensional molecular liquids,^187,188^ Nagao and colleagues estimated the membrane viscosity of two-dimensional lipid bilayers. The calculated membrane viscosity using the relaxation time attributed to the lipid molecular rearrangements (slower mode in the fluid phase) fell in the middle of the broadly distributed values for membrane viscosity reported in the literature, and the authors concluded that the lipid molecular rearrangements within the membrane were the origin of the membrane viscosity.^184^ This study opened up a new strategy to identify the molecular origins of the membrane viscosity.

## VI. SUMMARY AND OUTLOOK

Here, we reviewed measurements of the individual and collective lipid dynamics measured with neutron scattering techniques. The studied dynamics include lipid molecular rotational and translational diffusion within the membrane, acyl tail dynamics, as well as the collective undulation and thickness fluctuations of the membranes. These motions occur at different length and time scales from sub Å to tens of nm in length and sub ps to sub *μ*s in time scales that are accessible with neutron scattering techniques. The effects of different lipid compositions and various additives on the membrane dynamics have been studied by various groups, and the number and complexity of such membrane studies continue to grow. As the cell membrane is complicated, more detailed understanding of the molecular mechanisms of bilayer formation, stability, and functionality is demanded. The current efforts toward understanding the dynamics in model lipid membranes are essential as studies move to more and more complex systems, specifically, the work reviewed here lays the foundation for understanding future studies of lipid–protein interactions. These studies may also help connect lipid molecular movements to the elastic and viscous properties of lipid bilayers in the future.

Although the spatiotemporal capabilities of neutron scattering instruments well suited for the study lipid membrane dynamics, the number of neutron spectrometers is limited. Increasing the amount of beam time by increasing the number of instruments or increasing the neutron flux will help accelerate to understanding molecular mechanisms of membrane dynamics. Moreover, recent developments in neutron spectroscopy techniques further enhance the accessible dynamic ranges. For QENS studies, the development of the high resolution BS spectrometer IN16B enhances the highest energy resolutions compared to those of conventional BS instruments.^189^ Recent advances of NSE technology is also enhancing the time resolution of the technique, and it is now possible to extend the measurement times up to 1 *μ*s on state of the art NSE instruments.^18,190^ While the ns time range is sufficient to observe thermal fluctuations in model systems, many biological processes occur at time scales beyond thermal fluctuations, and extending the time resolution of the NSE technique to even longer time scales will allow access to such processes in the future. Another interesting development in the QENS field is the efforts to separate the coherent and incoherent scattering contributions by employing polarization analysis.^191–194^ Polarization analysis enables precise separation of the collective and self-motions^195,196^ and will be highly beneficial for lipid membrane studies. Most importantly, the neutron spectroscopy techniques reviewed here are accessible to general users through a proposal program at each facility worldwide. We encourage interested readers to contact instrument scientists at your facility of preference and develop ideas for new theories and experiments.

## Data Availability

Data sharing is not applicable to this article as no new data were created or analyzed in this study.

## NOMENCLATURE

**Instrumentation**
BS: backscattering
DLS: dynamic light scattering
DSC: differential scanning calorimetry
DSF: dynamic structure factor
EISF: elastic incoherent structure factor
GINSE: grazing incidence neutron spin echo
INS: inelastic neutron scattering
ISF: intermediate scattering function
MD: molecular dynamics
MTDI: Mössbauer time domain interferometry
NMR: nuclear magnetic resonance
NR: neutron reflectometry
NSE: neutron spin echo
QENS: quasielastic neutron scattering
SANS: small-angle neutron scattering
SAXS: small-angle x-ray scattering
TOF: time-of-flight
XDS: x-ray diffuse scattering

**Membrane theories**
BSO: Bingham, Smye and Olmsted
SL: Seifert and Langer
WB: Watson and Brown
ZG: Zilman and Granek

**Chemicals**
chol: cholesterol
CL: cardiolipin
C_12_E_5_: pentaethylene glycol monododecyl ether
DLPC: dilauroyl-phosphocholine
DMPC: dimyristoyl-phosphocholine
DMPG: dimyristoyl-phosphoglycerol
DOPC: dioleoyl-phosphocholine
DOPG: dioleoyl-phosphoglycerol
DOTAP: dioleoyl-trimethylammonium-propane
DPPC: dipalmitoyl-phosphocholine
DSPC: distearoyl-phosphocholine
ESM: egg sphingomyelin
G_*M*1_: ganglioside GM1
PAzPC: palmitoyl-azelaoyl-phosphocholine
PC: phosphocholine
PE: phosphoethanolamine
PG: phosphoglycerol
POPC: palmitoyl-oleoyl-phosphocholine
POPE: palmitoyl-oleoyl-phosphoethanolamine
POPG: palmitoyl-oleoyl-phosphoglycerol
SDS: sodium dodecylsulphate
SMPC: stearoyl-myristoyl-phosphocholine
SoyPC: L-*α*-phosphocholine

**General**
EVALI: electronic-cigarette/vaping-associated lung injury
HIV: human immunodeficiency virus
LUV: large unilamellar vesicle
MLV: multilamellar vesicle
NSAID: nonsteroidal anti-inflammatory drug
SARS-CoV: severe acute respiratory syndrome-associated coronavirus
ULV: unilamellar vesicle
